# Shift in Natural Groundwater Bacterial Community Structure Due to Zero-Valent Iron Nanoparticles (nZVI)

**DOI:** 10.3389/fmicb.2019.00533

**Published:** 2019-03-19

**Authors:** Marc Crampon, Catherine Joulian, Patrick Ollivier, Mickaël Charron, Jennifer Hellal

**Affiliations:** Bureau de Recherches Géologiques et Minières, Orléans, France

**Keywords:** zero-valent iron nanoparticles (nZVI), groundwater, nitrate-reducing bacterial community, ecotoxicity, remediation

## Abstract

Toxic and persistent contaminants in groundwater are technologically difficult to remediate. Remediation techniques using nanoparticles (NPs) such as nZVI (Zero-Valent Iron) are applicable as *in situ* reduction or oxidation agents and give promising results for groundwater treatment. However, these NP may also represent an additional contamination in groundwater. The aims of this study are to assess the impact of nZVI on the nitrate-reducing potential, the abundance and the structure of a planktonic nitrate-reducing bacterial community sampled in groundwater from a multicontaminated site. An active nitrate-reducing bacterial community was obtained from groundwater samples, and inoculated into batch reactors containing a carbon substrate, nitrate and a range of nZVI concentrations (from 0 to 70.1 mg Fe.L^-1^). Physical (pH, redox potential), chemical (${\mathrm{NO}}_{3}^{-}$ concentrations) and biological (DNA, RNA) parameters were monitored during 1 week, as well as nZVI size distribution and mortality of bacteria. Nitrate-reducing activity was temporally stopped in the presence of nZVI at concentrations higher than 30 mg L^-1^, and bacterial molecular parameters all decreased before resuming to initial values 48 h after nZVI addition. Bacterial community composition was also modified in all cultures exposed to nZVI as shown by CE-SSCP fingerprints. Surprisingly, it appeared overall that bacteria viability was lower for lower nZVI concentrations. This is possibly due to the presence of larger, less reactive NP aggregates for higher nZVI concentrations, which inhibit bacterial activity but could limit cell mortality. After 1 week, the bacterial cultures were transplanted into fresh media without nZVI, to assess their resilience in terms of activity. A lag-phase, corresponding to an adaptation phase of the community, was observed during this step before nitrate reduction reiterated, demonstrating the community’s resilience. The induction by nZVI of modifications in the bacterial community composition and thus in its metabolic potentials, if also occurring on site, could affect groundwater functioning on the long term following nZVI application. Further work dedicated to the study of nZVI impact on bacterial community directly on site is needed to assess a potential impact on groundwater functioning following nZVI application.

## Introduction

Due to their specific properties and high specific surface area, engineered nanoparticles (NP) are increasingly produced and used, leading to higher releases in the environment (Buzea et al., 2007). In particular, use of nanotechnology for environmental applications, such as remediation of contaminated groundwater, have steadily increased (Bardos et al., 2018). Among NP, nanoscale Zero-valent Iron (nZVI) particles are one of the most widely used NP for nanoremediation, because of their capacity to degrade a wide range of contaminants (Zhang, 2003; Zhang and Elliott, 2006; Mueller et al., 2012). Zero-valent iron (ZVI) is efficient for the dechlorination of PCE because of its ability to dehalogenate chlorinated compounds by chemical reduction (Fu et al., 2014). ZVI can be used in groundwater in permeable reactive barriers (PRB), that show effective degradation, but this technique is expensive, invasive and constrained by installation limits (Obiri-Nyarko et al., 2014). In this framework, an interesting alternative, can be direct injection of ZVI NPs into the aquifer, enabling the treatment of both source and plume areas (Cundy et al., 2008; Thiruvenkatachari et al., 2008; Stefaniuk et al., 2016). However, when directly injected into an aquifer, nZVI widely interact with bacteria and bacterial biofilms (Goldberg et al., 2007), which may affect nZVI mobility and reactivity. These NP may also present a toxicity for groundwater bacteria. Bacteria present in groundwater are relevant and sensitive indicators of soil or groundwater perturbations because of their key role in biogeochemical cycling (Brookes, 1995; Kandeler et al., 1996; Holden et al., 2014). They can be particularly important in the case of contaminated groundwater because of their ability to metabolize a wide range of contaminants (Antizar-Ladislao and Galil, 2010; Weaver et al., 2015; Zhao et al., 2016). Thus if nZVI application affects bacterial communities then it will also affect biogeochemical reactions occurring in the subsurface.

Several mechanisms of nZVI toxicity towards living organisms have been identified. The first is direct nZVI contact with biological components (O’Carroll et al., 2013). In the majority of studies that used electron microscopy to evaluate damage to cell integrity, precipitation of nZVI or iron oxides on the cell wall or inside the bacterial cells was commonly observed, suggesting that direct contact of nZVI with bacterial cells is required for nZVI to exert toxicity (Auffan et al., 2008; Diao and Yao, 2009; An et al., 2010; Barnes et al., 2010; Li et al., 2010; Xiu et al., 2010; Sevcu et al., 2011; Wille et al., 2017; Jiang et al., 2018; Xue et al., 2018). The second is the release of reactive oxidative species (ROS), such as hydroxyl radicals, superoxide radicals and hydrogen peroxide (Barnes et al., 2010), generated by nZVI in the aqueous phase (Liu et al., 2014). ROS are normally scavenged by antioxidants and various enzymes; however, elevated concentrations of ROS in microbial cells can result in oxidative stress. The damages caused to DNA in the presence of various organisms, including bacteria, were observed in other studies (Sacca et al., 2014; Ghosh et al., 2017). It has also been shown that some bacteria are able to produce proteins that protect them from oxidative stress caused by nZVI (Chaithawiwat et al., 2016; Xue et al., 2018). When nZVI is added into an aqueous medium, a burst of oxidants is produced as Fe0 and ferrous iron (Fe[II]) are converted to ferric iron (Fe[III]). Finally, the release of ferrous iron from nZVI followed by the Fenton reaction can also affect organisms (Xie and Cwiertny, 2012). Furthermore, although the mechanism of nZVI toxicity at the cellular level has not been fully determined, recent studies support the combined effect of biophysical nanoparticle/cell interactions and oxidative stress (Li et al., 2010; Wille et al., 2017; Crampon et al., 2018).

Impact of nZVI towards bacteria has been considerably studied (Jang et al., 2014), sometimes highlighting positive impacts (Wei et al., 2012; Kocur et al., 2016; Dong et al., 2019) and sometimes negative ones (Fajardo et al., 2013; Sacca et al., 2014; Dong et al., 2019). However, these studies generally focus on isolated species rather than addressing the complexity of natural groundwater bacterial communities (Lefevre et al., 2016) although toxicity of xenobiotics may be affected by interactions between the different members of a community. It is thus important to study their impact at the community scale. Indeed, inside a community, the degradation processes are often the result of synergistic degradation or cooperative metabolic interactions (Dejonghe et al., 2003; Freilich et al., 2011), especially in the case of multi-contamination. All members of the community may thus have a key-role in the degradation process. As it has been shown that nZVI may inhibit some groups but not the others inside a bacterial community (Kirschling et al., 2010; Xiu et al., 2010; Fajardo et al., 2013; Xue et al., 2018; Dong et al., 2019), it is important to characterize the toxicity at the scale of the whole community.

In this context, this study aims to deepen our understanding of the impact of nZVI towards groundwater bacterial communities by monitoring the impact of a gradient of nZVI concentrations on the nitrate-reducing potential, the abundance and the structure of a planktonic nitrate-reducing bacterial community. The experimental design was set up in order to monitor bacterial community resistance then resilience after being exposed to nZVI.

## Materials and Methods

### Experimental Design

**FIGURE 1:**
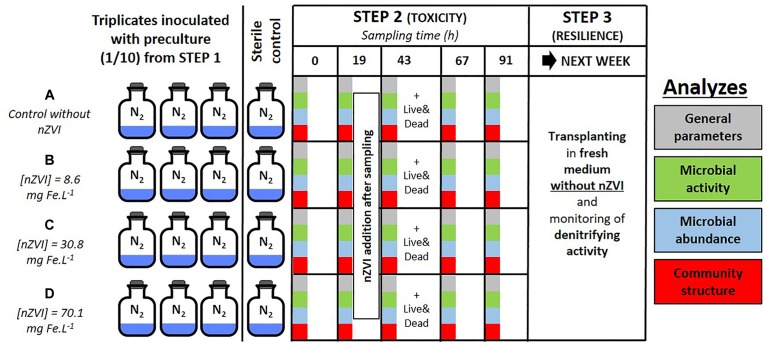
Experimental setup of the study with colors corresponding to the analyses on microbial communities: green for activity parameters, blue for abundance, and red for structural parameters. Panel **(A–D)** correspond to the different nZVI concentrations; these letters are used throughout the paper to discuss the different concentrations.

Both the toxicity of nZVI towards bacteria and the resilience of bacteria were studied by using a series of batch experiments. Three distinct steps were carried out and are illustrated in Figure 1. Step 1 consisted in enriching a heterotrophic nitrate-reducing community from a natural groundwater collected from an industrial site by adding a carbon substrate [10 mM sodium acetate (C_2_H_3_NaO_2_)], yeast extracts at 0.2 g L^-1^ and sodium nitrate (NaNO_3_) to a volume of groundwater (v/v) and monitoring nitrate reduction (Wille et al., 2017). Total nitrate reduction was obtained after about 48 h, then experiment proceeded to step 2. Step 2 was carried out for almost 5 days and consisted in two phases. The first one was the inoculation of the pre-culture at a ratio of 1/10 into hermetically sealed 250 mL Schott^®^flasks containing 180 mL of synthetic water enriched with a carbon substrate (10 mM sodium acetate), yeast extracts at 0.2 g L^-1^ and sodium nitrate, for a final volume of 200 mL. The second phase started after 19 h incubation in the dark at 20°C under agitation and confirmation of triggered nitrate reduction, when nZVI was added to triplicate flasks in order to expose the bacteria to a gradient of nZVI amounts: condition A (control without nZVI), B (8.6 mg Fe.L^-1^), C (30.8 mg Fe.L^-1^), and D (70.1 mg Fe.L^-1^). The addition of nZVI was carried out in an anaerobic chamber under a continuous flow of nitrogen (Figure 1). A sterile control without the bacterial inoculum was set up for each condition. 12 mL of liquid samples were collected daily up to 91 h incubation (at 0, 19, 43, 67, and 91 h, samples called T0, T19, T43, T67, and T91, respectively), and analyzed for chemical and biological parameters as described below. nZVI were added to the cultures after the T19 sampling. The third and last step of the experiment consisted of re-inoculating bacterial communities from conditions A, B, C, and D into fresh media (ratio 1/10, synthetic water in the same conditions as step 2) without nZVI to assess community resilience. These new batches were monitored for 3 days until all nitrate was reduced (samples collected at 0, 15, and 39 h incubation).

The synthetic water used in these experiments is FIm water corresponding to moderately hard water as described in US EPA Report EPA-821-R-02-012, Section 7.2.3.1 (US EPA, 2002). This water was prepared as previously described in Crampon et al. (2018), and was composed of 96 mg L^-1^ NaHCO_3_, 60 mg L^-1^ CaSO_4_.2H_2_O, 60 mg L^-1^ MgSO_4_, and 4 mg L^-1^ KCl. The pH ranged from 7.4 to 7.8, the hardness ranged from 80 to 100 mg CaCO_3_.L^-1^ and the alkalinity from 57 to 64 mg CaCO_3_.L^-1^ as described by US EPA. The synthetic water was purged with N_2_ to remove any oxygen prior to culture medium preparation.

In this experimental setup, as a model activity for the groundwater bacterial community, we focused on bacterial nitrate reduction. This was assessed by measuring nitrate depletion and the abundance of the *narG* gene and its expression. The overall bacterial community was also characterized by determining abundance (qPCR 16S rRNA), structure (CE-SSCP), composition (Cloning/Sequencing) and viability (LIVE&DEAD^®^). Physico-chemical parameters were also followed such as pH, redox potential (ORP) and NP size. All analytical approaches are described in the following sections.

#### Physico-Chemical Analyses

Fe concentrations were measured by ICP-AES (Jobin Yvon Horiba Ultima 2; Horiba, Japan) in water samples from the cultures at the beginning of the experiment, after acidification with HCl (to obtain a pH < 2). Presented values correspond to the means of two culture replicates for each range of nZVI concentration. Obtained Fe concentrations were 8.6 ± 1.4 mg Fe.L^-1^ (*n* = 2) for the lower concentrations (condition B), 30.8 ± 10.2 mg Fe.L^-1^ for intermediate concentrations (condition C) and 70.1 ± 14.8 mg Fe.L^-1^ for higher concentrations (condition D).

Due to the presence of ions in the culture medium, NP aggregates were expected to form. The size and zeta potentials of these aggregates were consequently measured. The size of NP aggregates was measured using Dynamic Light Scattering (DLS, Malvern Zetasizer Nano ZS, Malvern, United Kingdom) in cultures at the end of step 2. It was not possible to distinguish here homo- (formed by nZVI aggregation) from hetero- aggregates (formed by aggregation of nZVI with bacteria and/or biofilm). Electrophoretic mobility (EPM) was measured and the apparent zeta potential at 25°C was calculated automatically by Zetasizer software using the Smoluchowski equation (Hunter, 2001). The polydispersity index (PdI) was used as an indicator of the distribution width. A lower PdI corresponds to a lower sorting of NP, due to the presence of a higher amount of aggregates in solution.

Measurements of pH and redox potential (ORP) were performed for each sampling point in an anaerobic chamber under N_2_ atmosphere. Measurements were done with a VTW Multi parameter unit (VTW/Xylem, Inc., Germany) with specific pH and ORP electrodes.

#### Nitrate Reduction Activity Measurements

The determination of nitrate concentrations was performed on filtrated water (10 mL, filtration through a 0.22 μm mixed cellulose esters membrane GSWP, Merck Millipore). The analyses were performed at T0, and after 19, 43 h (i.e., 24 h after nZVI addition), 67 and 91 h growth during step 2 and at T0 and after 15 and 39 h growth during step 3. The concentrations of NO_3_-N were determined by spectrophotometry using a Nova 60 Spectroquant spectrophotometer (Merck/Millipore, Germany) with a specific Spectroquant nitrate kit (reference 114563, Merck/Millipore). The range of concentrations of the kits was 0.1 to 25 mg L^-1^ of NO_3_-N. We decided to focus here on nitrate measurements rather than on the reduction product nitrite as it was only transient and most often below limits of quantification when measured.

### Characterization of Bacterial Communities

#### LIVE/DEAD^®^ BacLight^TM^ Viability Assays

LIVE/DEAD^®^ BacLight^TM^ Bacterial Viability Kits were used according to the manufacturer’s instructions (Invitrogen, Carlsbad, CA, United States). In brief, a volume of reactive containing a mixture of SYTO 9 dye (green-fluorescent nucleic acid stain) and Propidium iodide (red-fluorescent nucleic acid stain) is added to an equal volume of culture medium, mixed then incubated in the dark at room temperature for 15 min. Cells are then observed and counted using a fluorescent microscope with filters in the range of 480/500 nm for SYTO 9 stain and 490/635 nm for propidium iodide. SYTO 9 dye tends to stain all bacteria in a population whereas propidium iodide only penetrates cell walls of damaged bacteria, thus showing “live” bacteria as green and “dead” bacteria as red. Observations were made on samples from step 2, at T43, i.e., 24 h after nZVI addition. Preliminary tests (data not shown) were performed on the bacterial community at nZVI concentrations of 10, 30, and 50 mg Fe.L^-1^ to perform counts of living and dead cells.

#### DNA and RNA Extractions and cDNA Generation

Extractions of DNA and RNA were performed on samples before nZVI addition (T19), after nZVI addition (T43, i.e., 24 h after nZVI addition) and after 67 h incubation (T67, i.e., 48 h after nZVI addition). Biomass was recovered by filtering 10 mL culture on 0.22 μm filters (mixed cellulose esters membrane GSWP, Ø 25 mm, Merck Millipore).

Genomic DNA and total RNA were co-extracted directly from the filters with the combination of MO Bio/QIAGEN (Valencia, CA, United States) kits. First, RNA was extracted with Mo Bio RNeasy^®^ PowerSoil^®^ Total RNA Kit, and DNA was then recovered thanks to the DNA Elution Accessory Kit, according to the manufacturer’s instructions. Remaining DNA was removed from RNA extracts with the turbo DNase (Ambion, Carlsbad, CA, United States) according to the manufacturer’s instructions, and RNA was resubmitted to the purification step of the RNeasy^®^ PowerSoil^®^ Total RNA Kit. The absence of DNA in RNA extracts was confirmed by no PCR signal (see below for PCR conditions). Quantifications of extracted RNA and DNA were performed with the Quantus Fluorometer, using 1 μL extract and the QuantiFluor dsDNA solution (Promega, Charbonnières-les-Bains, France).

The cDNA were obtained from 50 ng RNA extracts by RT-PCR performed on with iScript^TM^ cDNA synthesis kit and iScript^TM^ Reverse Transcription Supermix kit (Biorad), following the manufacturer’s instructions.

DNA and cDNA were stored at -20°C and RNA at -80°C.

#### qPCR of 16S rRNA and *narG* Genes

Quantification of the bacterial 16S rRNA and *narG* gene copies was performed by qPCR using a CFX Connect^TM^ Real-Time PCR Detection System (Bio-Rad, France) with primers 341F (3′-CCTACGGGAGGCAGCAG-5′) and 515R (3′-ATTACCGCGGCTGCTGGCA-5′) (López-Gutiérrez et al., 2004) for 16S rRNA gene, and primers narG-F (5′-TCGCCSATYCCGGCSATGTC-3′) and narG-R (5′-GAGTTGTACCAGTCRGCSGAYTCSG-3′) for nitrate reductase *narG* gene carried by *Proteobacteria* (Bru et al., 2007), as only *Proteobacteria* developed in the batch experiments. qPCR reactions were performed in a total volume of 20 μL, with a master mix containing 7.6 μL of sterile, nuclease- and nucleic acids-free water (MP Biomedicals, Santa Ana, CA, United States), 10 μL of SSO Advanced Universal SYBR Green Supermix (Bio-Rad), 500 nM of each primer, 100 ng of T4 GP32, and 2 μL of template DNA (0.1 to 5 ng μL^-1^) or 2 μL of cDNA. Sterile, nuclease- and nucleic acids-free water was added instead of DNA in no template controls. PCR reactions were performed as follows: 3 min 95°C, followed by 35 cycles: 30 s 95°C/30 s 60°C/30 s 72°C/for 16S rRNA gene or 6 cycles: 30 s 95°C/30 s 63°C/30 s 72°C, 34 cycles: 30 s 95°C/30 s 58°C/30 s 72°C for *narG* gene, followed by 30 s at 80°C for data acquisition, with an additional step rising from 60 to 95°C at 0.5°C/s for melt curves generation. All samples, controls and standards were analyzed in duplicates. A calibration curve was obtained from serial dilutions of a known quantity of linearized plasmids containing known copy numbers of 16S rRNA or of *narG* gene. Results were reported as gene copies per mL of culture medium. Generation of a specific PCR product was confirmed by melting curve analyses and agarose gel electrophoresis.

#### CE-SSCP Fingerprints

The bacterial community structure in cultures during the experiment was determined by 16S rRNA gene CE-SSCP fingerprints. About 200 bp of the V3 region of bacterial 16S rRNA genes were amplified from DNA and cDNA with the forward primer w49 (5′-ACGGTCCAGACTCCTACGGG-3′; *Escherichia coli* position, 331) and the reverse primer w34 (5′-TTACCGCGGCTGCTGGCAC-3′; *E. coli* position, 533), as previously described in Crampon et al. (2018). The 5′ end was then labeled with the fluorescent dye FAM. 25 cycles and hybridization at 61°C were used. One μL of diluted (150-fold in nuclease-free water) PCR product was added to a mixture of 18.8 μL of deionized formamide and 0.2 μL of Genescan-600 LIZ internal standard (Life Technologies, Carlsbad, CA, United States). To obtain single-strand DNA, samples were heat denaturated for 5 min at 95°C, and immediately cooled on ice. CE-SSCP analyses were performed on an ABI Prism 310 genetic analyzer using a 47-cm length capillary, a non-denaturating 5.6% CAP polymer (Life Technologies) and the following electrophoresis conditions: run temperature 32°C, sample injection for 5 s at 15 kV, data collection for 35 min at 12 kV. The dataset was analyzed using the software BioNumerics V7.5 (Applied Maths) and lining fingerprints up to the internal standard and to a common baseline.

#### Bacteria Taxonomic Identification

About 1,400 bp of the bacterial 16S rRNA gene were amplified from the DNA and cDNA. Universal bacterial primers 8F (5′-AGAGTTTGATCMTGGCTCAG-3′) and 1406R (5′-ACGGGCGGTGTGTRC-3′) were used with hybridization at 55°C and 30 cycles. Purified PCR products (NucleoSpin^®^ Gel and PCR clean-up, Macherey–Nagel) were used to construct a gene library with the TOPO-TA Cloning Kit and TOP 10 chemically competent cells (Invitrogen, Carlsbad, CA, United States). To ensure good characterization of the dominant strains detected by CE-SSCP, positive transformants were analyzed by CE-SSCP to find correspondence of clones over the whole community. Based on their migration profile, seven clones were selected for sequencing the insert with vector primers T7 and T3 (Sanger sequencing by GATC Biotech). All seven sequences were submitted to NCBI GenBank under Accession Nos. MH686140 to MH686146.

#### Phylogenetic Analysis

The phylogenetic assignment of unambiguous consensus sequences was based on comparison with reference sequences by the NCBI Blast tool^1^. Multiple alignment of sequences and consensus were performed using BioEdit software version 7.0.5.3 (Hall, 1999). The phylogenetic tree was constructed using the neighbor-joining method of Jukes and Cantor (Saitou and Nei, 1987), and checked by UPGMA method included in MEGA 10 package (version 10.0.1). The tree topology was tested by bootstrap analysis of 1,000 resamplings. An *E. coli* 16S rRNA gene sequence was used as an outgroup.

### Statistical Analyses

Statistical analyses were performed on XLStat software version 2017 (Addinsoft) for MS excel. Principal Coordinate Analyses were built from Pearson’s correlations coefficients similarity matrices, from triplicates values of the different analyzed parameters. As trends are not linear with nZVI concentrations (inversed trends), control samples were excluded from PCoA analyses.

## Results

### Physico-Chemical Parameters

The mean sizes of nZVI were measured at the end of the first week of the experiment, as well as ZP, in triplicates for each nZVI concentration. ZP of nZVI in cultures was logically negative, and ranged from -37.1 to -23.3 mV.

Globally, PdI was lower for higher nZVI concentrations [PdI = 0.45 ± 0.03 (*n* = 3) and 0.46 ± 0.08 (*n* = 3) for Fe concentrations of 30.8 and 70.1 mg Fe.L^-1^, respectively], meaning that a higher concentration of nZVI leads to a lower sorting of aggregates. PdI mean was 0.57 ± 0.11 (*n* = 3) for lower nZVI concentrations (i.e., 8.6 mg Fe.L^-1^), showing the presence of less aggregates of different sizes in cultures, and consequently the presence of a higher number of individualized NP.

Even if a trend is observed, showing a higher mean diameter for higher nZVI concentrations (Table 1), the mean sizes were not significantly different between the different concentrations (*P* > 0.05, Kruskal–Wallis test with Conover–Iman pair comparison). Concerning nZVI size sorting shown by PdI values (a value of 1 corresponding to a monodisperse sample, 0 to a polydisperse sample), no significant difference could be highlighted either between the different concentrations (*P* > 0.05, Kruskal–Wallis test with Conover–Iman pair comparison). However, a trend was again observed (Table 1), with a better sorting for lower nZVI concentrations.

**Table 1:** Values of Fe concentration, mean NP sizes, PdI and zeta potentials obtained by ICP-AES and DLS analyses in the different cultures.

		A *(Control without nZVI)*	B *([nZVI] = 8.6 mg Fe.L^-1^)*	C *([nZVI] = 30.8 mg Fe.L^-1^)*	D *([nZVI] = 70.1 mg Fe.L^-1^)*
Fe concentrations	*mg.L^-1^*	0.0 ± 0.0	8.6 ± 1.4	30.8 ± 10.2	70.1 ± 14.8
NPs mean diameter	*nm*	*NA*	1094.5 ± 135.0	1076.0 ± 38.7	1307.0 ± 74.2
PdI	-	*NA*	0.572 ± 0.119	0.449 ± 0.037	0.405 ± 0.133
Zeta potential	*mV*	*NA*	-26.8 ± 0.6	-24.0 ± 1.0	-32.5 ± 6.5


*NA, not applicable.*

**FIGURE 2:**
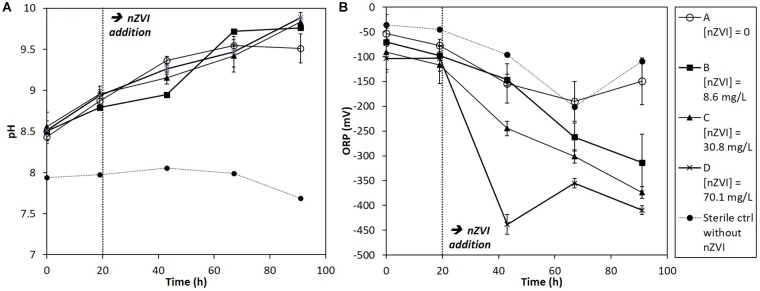
Monitoring of **(A)** pH and **(B)** ORP (mV) in batch experiments for all conditions (A, B, C, and D; see text for details) during 91 h of incubation. nZVI concentrations are expressed as total Fe concentrations.

Redox potential (ORP) and pH trends were measured in cultures at each sampling date (Figure 2). Globally, pH tended to increase during the experiment in all conditions due to the bacterial activity, whereas it was stable in sterile controls (Figure 2A). The pH of cultures rose up to around 10 at the end of the experiment. Concerning ORP, it greatly decreased, straight after nZVI addition, logically due to nZVI reduction properties (Figure 2B). The lowest ORP values were observed for the highest nZVI concentrations. No ORP decrease was observed just after nZVI addition for the lower concentration (B, 8.6 mg Fe.L^-1^) compared to the absence of nZVI and to higher nZVI concentrations, but ORP tended to decrease to about -300 mV after 91 h of incubation.

### Effects of nZVI on Microbial Abundance

#### Molecular Biomass

**FIGURE 3:**
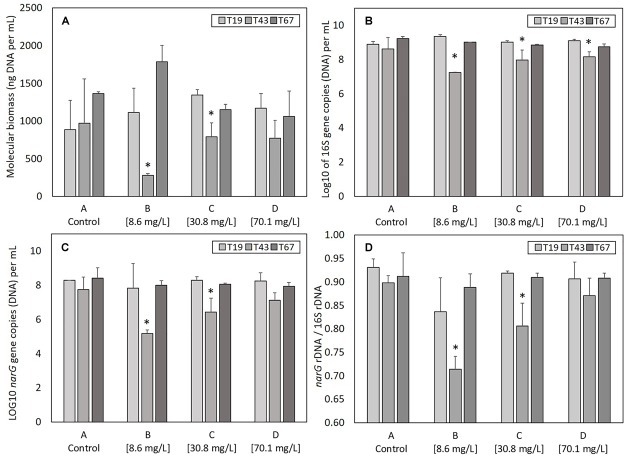
Parameters linked to bacterial abundance: **(A)** molecular biomass (genomic DNA concentration), **(B)** Log10 of 16S rRNA gene copies, **(C)** Log 10 of *narG* gene copies, and **(D)** ratio *narG* over 16S rRNA genes copies, at T19 (before nZVI addition) and T43/T67 (after nZVI addition). ^∗^ Corresponds to a significant difference (*P* < 0.05, Student’s *t*-test) between T19 and T43 values, corresponding to a significant impact of nZVI addition.

The total genomic DNA extracted from samples gives an indication of the total molecular biomass. Molecular biomass decreased after nZVI addition for all nZVI concentrations (T43; Figure 3A). The decrease was significant (*P* < 0.05, Student’s *t*-test) for conditions B (8.6 mg Fe.L^-1^) and C (30.8 mg Fe.L^-1^), but not for the D modality (70.1 mg Fe.L^-1^). Moreover, the decrease in molecular biomass was higher for condition B compared to condition C. These results suggest that the highest impact on molecular biomass occurs for lower nZVI concentrations. Forty-eight hours after exposure (T67), molecular biomass increased and reached control values, suggesting a resilience of microorganisms in the community. Surprisingly, at T67, the highest molecular biomass was measured in condition B, where the initial impact on molecular biomass, measured at T43, was highest.

#### Bacterial Abundance

As for molecular biomass, nZVI had a significant impact on bacterial abundance, as shown by the decrease in the number of copies of the 16S rRNA gene at T43 in cultures exposed to nZVI in conditions B, C, and D (Figure 3B). This impact was significant (*P* < 0.05, Student’s *t*-test) for all modalities. However, the highest impact of nZVI was again observed for lower concentrations (i.e., 8.6 mg Fe.L^-1^). Finally, 48 h after exposure (T67), the abundance of the 16S rRNA gene was comparable to values measured in control samples, again suggesting a resilience of the bacterial community.

#### Nitrate-Reducing Bacteria Abundance

The determination of *narG* gene copies gives an indication of the abundance of nitrate-reducing bacteria in the bacterial community. As was observed for molecular biomass (genomic DNA concentration) and bacterial abundance (numbers of 16S rRNA gene copies), a decrease in the abundance of nitrate-reducing bacteria occurred after nZVI addition (T43) independently from nZVI concentrations (Figure 3C). This decrease was only significant for conditions B and C (8.6 and 30.8 mg Fe.L^-1^, respectively, *P* < 0.05, Student’s *t*-test). After 48 h of exposure (T67), the abundance of nitrate-reducing bacteria was comparable to those of the controls. Again, the lowest concentration of nZVI showed the highest impact, but the nitrate-reducing bacteria were resilient, in terms of biomass, after 48 h exposure.

Calculating the ratio between *narG* and 16S rRNA genes gives an indication of nZVI impact specifically on nitrate-reducing bacteria over the whole community (Figure 3D). Before nZVI addition, about 90% of total bacteria carried the *narG* gene. This value is significantly lower after nZVI addition for conditions B and C, especially for the lowest nZVI concentration. This value drops to around 70% of nitrate-reducing bacteria in the whole community for condition B, and 80% for condition C. The ratio of nitrate-reducing bacteria is the same for condition D (70.1 mg Fe.L^-1^) compared to control. Overall, these parameters remained stable for the control condition A.

#### Bacteria Viability

**FIGURE 4:**
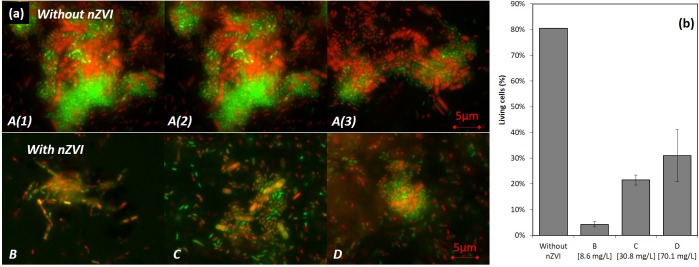
LIVE/DEAD^®^ observations (illustration images) **(a)** and results of counting **(b)** of bacteria after 43 h of incubation (24 h after nZVI addition). A [A(1), A(2), and A(3)] images are for control samples where huge living cells aggregates were observed, and B/C/D are images of cultures containing nZVI. Please note that images from **(a)** were not those used for LIVE/DEAD counting results presented in **(b)**.

In the control condition A (no nZVI), many live bacteria aggregates were observed after 43 h of incubation [T43; A(1), A(2), and A(3) in Figure 4a,A]. However, with nZVI (Figure 4a,B–D), bacterial aggregates were not found, except smaller ones in condition D, and more dead cells were present.

A viability of around 80% in the control condition was observed (counts were performed on diluted samples with between 10 and 50 cells per image). This value drops to around 5% in condition B, 20% in condition C and 40% for the highest concentration in condition D (Figure 4b). The higher toxicity in the presence of lower concentrations of nZVI is thus again confirmed here by LIVE/DEAD^®^observations, with a smaller number of living cells in the case of lower nZVI concentrations.

### Effects of nZVI on Microbial Activity

#### Nitrate Reduction Kinetics

**FIGURE 5:**
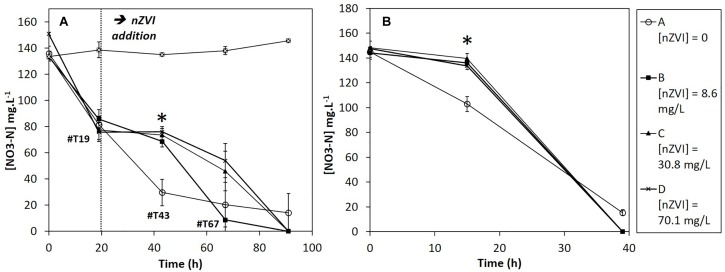
Evolution of nitrate concentrations during **(A)** Step 2: the first week experiment in the presence of nZVI (exposure) and **(B)** Step 3: after re-inoculation in fresh media without nZVI (resilience). ^∗^ Corresponds to a significant difference between control and cultures with nZVI.

The nitrate concentrations in sterile controls remained stable all along the experiment. Moreover, nZVI had no significant impact on nitrate concentrations (Supplementary Material SM2). Therefore, the observed nitrate reduction in cultures is of bacterial origin. Before nZVI addition (Figure 5A), nitrate reduction occurred and was repeatable for all conditions. After nZVI addition, nitrate reduction stopped in all cultures, except in control without nZVI. However, 24 h after nZVI addition (T43), nitrate reduction activity was again observed in all cultures with nZVI. After 91 h, remaining nitrate concentrations were even lower than in cultures without nZVI. These results highlight a resilience capability of the nitrate-reducing bacterial community after exposure to nZVI, even if nZVI initially had a significantly toxic impact on nitrate reduction (*P* > 0.05, Kruskal–Wallis test with Conover–Iman pair comparison).

During the resilience step (second week), nitrate reduction was monitored until complete nitrate disappearance (Figure 5B). For all cultures previously exposed to nZVI during the first week experiment, a 15-h lag-phase was observed. The difference was significant between cultures that had not been exposed to nZVI (A), for which no lag phase was observed, and cultures that had been exposed to nZVI (B, C, and D), independently from nZVI concentrations (*P* > 0.05, Kruskal–Wallis test).

Of note, in complement to sterile controls presented here, a preliminary experiment was led, with nZVI concentrations ranging from 1 to 500 mg Fe.L^-1^ in sterile conditions and with comparable NO_3_ concentrations, to study the impact of nZVI on nitrates (Supplementary Material SM1). The presence of nZVI did not impact nitrate concentrations, independently from nZVI concentrations.

#### Bacterial Activity: RNA vs. DNA

**FIGURE 6:**
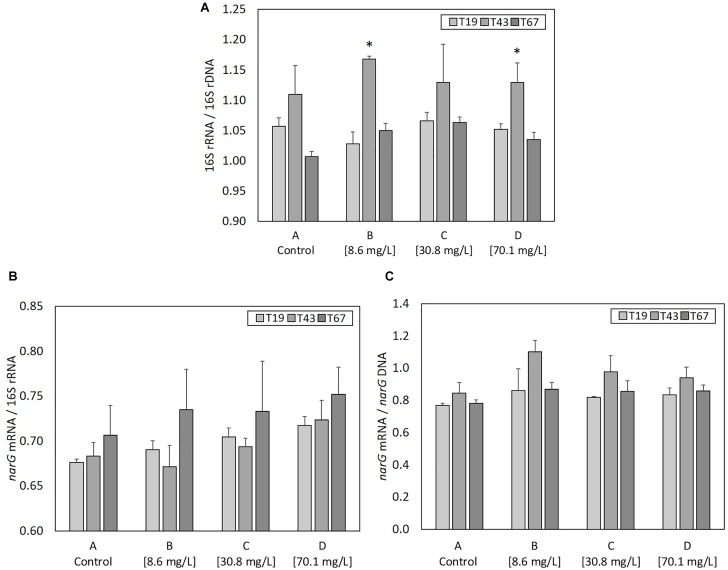
Parameters linked to bacterial activity at T19 (before nZVI addition) and at T43/T67 (i.e., after nZVI addition): **(A)** 16S rRNA/16S rDNA (bacterial community growth state), **(B)** *narG* mRNA/16S rDNA (nitrate reduction activity of the bacterial community), and **(C)** *narG* mRNA/*narG* DNA (nitrate reduction activity of nitrate-reducing bacteria over the bacterial community). ^∗^ Corresponds to a significant difference (*P* < 0.05, Student’s *t*-test) between T19 and T43 values, illustrating a significant impact of nZVI addition.

As ribosome content (rRNA) in bacterial cells is an indicator of active or recently active bacteria, ratios of 16S rRNA copy numbers (determined from cDNA generated from total RNA) to 16S rRNA gene copy numbers (determined from DNA) was used to evaluate the growth state of the bacterial community (Figure 6A). 16S rRNA/16S rDNA ratio was highest 24 h after nZVI addition (T43) compared to before addition (T19) in all conditions. It was significantly higher (*P*-value < 0.05) for conditions B and D, and, although statistically un-significant, the same trend was observed for conditions A and C. Thus, despite a negative effect on microbial biomass and bacterial abundance, nZVI did not affect the global growth state of bacteria remaining in the community.

#### Nitrate Reduction Activity: *narG* mRNA/16S rRNA and *narG* mRNA/*narG* DNA

*narG* transcripts (*narG* mRNA) were used to evaluate the nitrate reduction activity. The ratio between the number of *narG* mRNA and 16S rRNA (Figure 6B) highlights the part of nitrate-reducing activity among active or recently active bacterial cells in the community. No significant changes were observed between controls and cultures containing nZVI or between samples collected just before (T19) and after (T43) nZVI addition. Therefore, the impact of nZVI on the nitrate reduction activity was not clearly highlighted here. Only trends were observed at T43, with a decrease in *narG* transcripts/16S rRNA ratios, suggesting a decrease in the part of nitrate reduction among active or recently active bacterial cells, and again for lower nZVI concentrations. The ratio of *narG* mRNA to *narG* DNA shows the nitrate reduction activity of the nitrate reduction community. No significant differences were observed between controls and conditions with nZVI, or between samples collected just before (T19) and 24 h after (T43) nZVI addition, suggesting that the remaining nitrate-reducing bacteria were active. Even if not significant, the *narG* mRNA/DNA ratio tends to increase at T43 for cultures with nZVI, suggesting that nZVI addition, especially at lower concentrations, slightly increased the activity of remaining nitrate-reducing cells (Figure 6C).

In order to compare the impact of the different nZVI concentrations on biological and physico-chemical parameters at the different times, statistical analyses were performed (PCoA, Supplementary Material SM3). No correlations between nZVI concentrations and the different studied parameters were observed, as expected, before nZVI addition (T19). After nZVI addition, the maximum correlation was observed at T43 (24 h after nZVI addition), but was no longer relevant at T67, indicating that the toxicity of nZVI, for these parameters, lasted for no more than 48 h.

### Effects of nZVI on Microbial Community Structure

**FIGURE 7:**
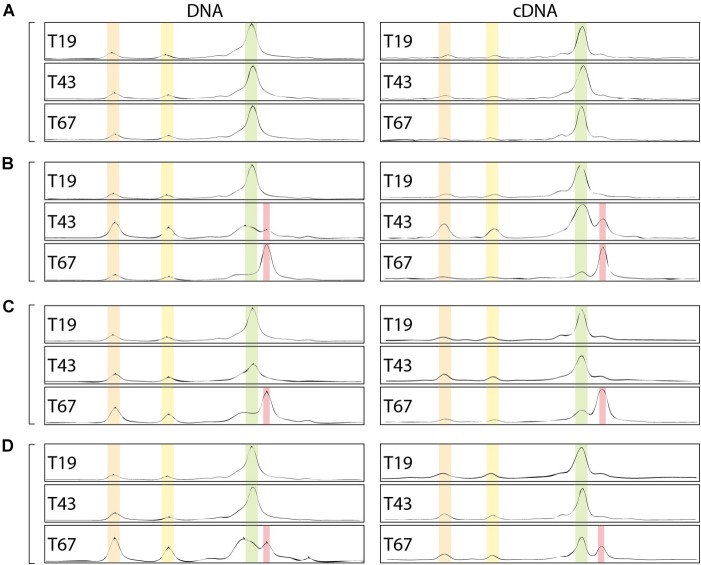
Bacterial community fingerprints assessed by CE-SSCP of 16S rRNA gene on DNA and RNA (cDNA). **(A–D)** Corresponds to the different nZVI concentrations, i.e., 0, 8.6, 30.8, and 70.1 mg Fe.L^-1^, respectively; T19: before nZVI addition, T43: 24 h after nZVI addition, T67: 48 h after nZVI addition.

Beyond the impact on abundance and activity, the bacterial community structure could also be affected by the presence of nZVI. Community CE-SSCP fingerprints of the 16S rRNA gene were performed on (i) DNA and (ii) cDNA, which allows to observe (i) the structure of the total community and (ii) the structure of active or recently active communities. Before nZVI addition, fingerprints showed three main peaks with one dominating the others, and suggested a low bacterial diversity in all conditions. Overall, fingerprints were similar for DNA and cDNA, highlighting a good adequacy between detection and growth state of all detected bacteria in the cultures. After nZVI addition, a clear shift of the bacterial community structure occurred. A new peak (in red) was detected to the detriment of the dominant peak (in green in Figure 7), which was highly affected by nZVI addition (Figure 7B–D).

Indeed, especially at T67, a shift in diversity was observed, with the detection of a new peak (in red in Figure 7) at the expense of the main peak (in green). This shift was more marked for lower nZVI concentrations (Figure 7B), where the new peak became dominant on the fingerprints (Figure 7). However, as nZVI concentrations increase, the new peak appears less and less dominant, and proportions between green and red peaks are quite similar for conditions D (Figure 7). After nZVI addition, the relative proportion of bacteria corresponding to peaks 1 and 2 increased in the community. This result is intermediate for condition C (Figure 7C). For condition B, the new peak in red appeared at T43, 24 h after nZVI addition, and became dominant at T67, 48 h after nZVI addition. Finally, the relative proportion of increasing bacteria, corresponding to the red peak, was the highest at T67, especially for B and C conditions for which it became the dominant peak. Globally, nZVI had an impact on the bacterial community structure, either favoring or affecting presence and abundance of the bacterial strains.

### Bacteria Identification

**FIGURE 8:**
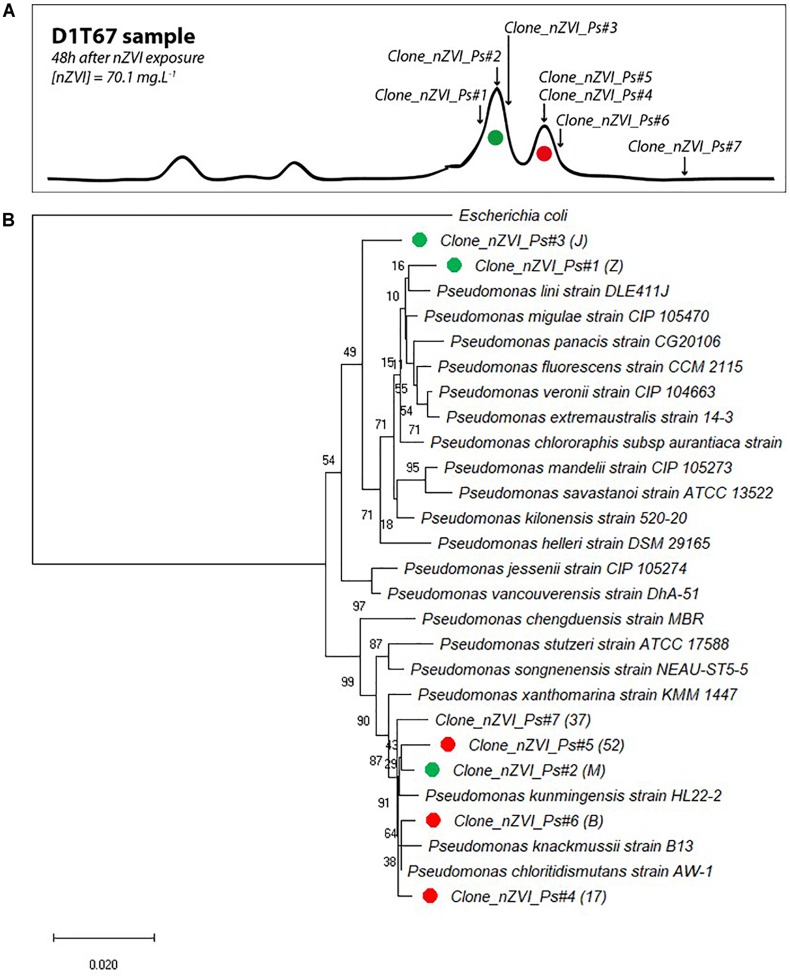
**(A)** Correspondence of sequenced clones in bacterial community fingerprints (here presented data from condition D 1 at T67). Green clones correspond to the first peak of interest and red clones to the second peak of interest (peak that appeared in the presence of nZVI). **(B)** Evolutionary relationships of taxa. The percentage of replicate trees in which the associated taxa clustered together in the bootstrap test (1000 replicates) are shown next to the branches. The tree is drawn to scale, with branch lengths in the same units as those of the evolutionary distances used to infer the phylogenetic tree.

Cloning and sequencing of community 16S rRNA gene at the end of the experiment (T67) and CE-SSCP migration patterns of retrieved clones allowed to attribute several sequences to the main peaks of the fingerprints (Figure 8A). All 16S rRNA gene clone sequences were closely related (>99% blast identity) to that of members of the *Pseudomonas* genus (Figure 8B). Species from the *Pseudomonas* genus are difficult to distinguish with 16S rRNA gene sequences, as they are very closely related to each other (Anzai et al., 2000). However, although we cannot clearly link a sequence to a species, phylogenetic comparison with close members of the *Pseudomonas* genus confirmed the shift in community composition. For the first peak of interest (in green), prior to nZVI addition, three clones were identified (*Clone_nZVI_Ps#1, #2*, and *#3*). In particular, *Clone_nZVI_Ps#1* branched among facultative anaerobic *Pseudomonas* that are able to perform nitrate reduction (Supplementary Material SM4). Consequently, *Pseudomonas* strains corresponding to the dominant green peak in the fingerprints could be the one able to perform nitrate reduction, and could be involved in the nitrate reduction process observed in the experiments. For the second peak of interest (in red), after nZVI addition, the three clones identified (*Clone_nZVI_Ps#4, #5*, and *#6*) are phylogenetically different from *Clone_nZVI_Ps#1* and *#3*. They are highly related to each other (dissimilarity < 0.01%), and very close to *Pseudomonas kunmingensis, Pseudomonas knackmussii*, and *Pseudomonas chloritidismutans* sequences. *Pseudomonas kunmingensis* is an aerobic bacteria able to perform nitrate reduction (Romanenko et al., 2005; Xie et al., 2014). *Pseudomonas knackmussii* and *Pseudomonas chloritidismutans* are facultative aerobic bacteria, unable to perform nitrate reduction (Supplementary Material SM4). However, as nitrate reduction then resumed, either the rising strain could reduce nitrate or nitrate reduction involved bacteria not detected by CE-SSCP. Indeed, one last clone sequenced (*Clone_nZVI_Ps#7*) also affiliated to *Pseudomonas*, did not correspond to a peak visible on the CE-SSCP fingerprint.

## Discussion

This work studied the impact(s) of nZVI addition on the abundance, the activity and the structure of a bacterial community grown from a natural groundwater. This was experimentally assessed by focusing on a key function in groundwater, nitrate reduction. Moreover, a follow-up experiment was carried out to look at the bacterial community’s reaction when inoculated in fresh culture medium after their exposure to nZVI.

Addition of nZVI, even at low concentrations of ∼9 mg L^-1^ significantly decreased molecular biomass and bacterial abundance 24 h after addition as shown by LIVE/DEAD^®^observations, molecular biomass and 16S rRNA and *narG* gene abundances. However, molecular biomass then increased again rapidly in all conditions suggesting only a temporary effect on bacterial abundance. Few studies have assessed the impact of nZVI in planktonic groundwater or water studies. Barnes et al. (2010) observed an increase in CFU counts in river water submitted to 100 mg L^-1^ nZVI with no initial decrease. Kirschling et al. (2010) also observed no impact of 1.5 g L^-1^ nZVI on the number of 16S rRNA gene copies in bacterial communities from TCE contaminated groundwater. However, this was shown after 250 days incubation so it is possible that the community had time to adapt. Fajardo et al. (2012) found little impact of 34 mg.nZVI.g^-1^ soil on microbial viability; however, as underlined by Fajardo et al. (2013) and Sacca et al. (2014), the impact of nZVI in soils is closely linked to their composition whereas in the present study, in an aqueous solution, bacterial communities would be more directly exposed, as it would be the case in aquifers (less complex compared to soils, low content of natural organic matter).

Similarly, the growth state and nitrate-reducing activity, assessed by the ratio of *narG* transcripts and nitrate depletion, were only affected temporally by the three concentrations of nZVI, suggesting an adaptation of the bacterial community to the new environment containing nZVI. The activity of remaining bacteria in condition B, the most affected by nZVI addition, even appeared to be stronger compared to the less impacted cultures. At the end of the experiment, no nitrates were left in nZVI-exposed cultures, whereas a residue was always found in controls without nZVI. One explanation could be anaerobic corrosion of nZVI, leading to the release of H_2_ in the media, a well-known electron donor for bacteria, reinforcing their metabolism and resulting, after an adaptation period, to a more complete nitrate reduction in the presence of nZVI. A lower nitrate concentration was also observed during the resilience step for cultures having been exposed to nZVI, and could be explained both by residual H_2_ introduced in fresh media by the inoculum (1/10 volume), or by the bacterial community structure modifications generated by nZVI during the first step of the experiment. Even if nitrate reduction activity resumed and if parameters related to bacterial abundance and activity returned to “normal” values, the structure of the bacterial community was modified by nZVI. Indeed CE-SSCP fingerprints revealed the increase of a new peak after nZVI addition, first in condition B which was the most impacted globally, and then in conditions C and D. This new peak then dominated the incubations and was confirmed to be a different strain to the initially dominating peak by sequencing fragments of 16S rRNA gene. These changes in the community structure due to nZVI were previously studied, and attributed both to H_2_ release and changes in redox conditions, favoring the growth of methanogen or sulfate-reducing bacteria (Kirschling et al., 2010; Xiu et al., 2010), but also to some specific nZVI coatings, protecting bacteria from oxidative stress, and creating a new carbon source in the media (Kirschling et al., 2010). In our case (non-coated nZVI), a rapid impact could be observed, attributable to toxic effect (oxidative stress), but the community structure was definitely modified even if nitrate-reduction resumed after a lag-phase This impact on community structure is highly dependant on the media, as was shown in soils (Fajardo et al., 2018). The variability of the media is however lower in groundwater compared to soils, and so changes in community structure due to nZVI are expected to occur more currently. These results are finally comparable to several previous experiments carried out either in water samples or soils. Indeed, both Fajardo et al. (2012) and Sacca et al. (2014) observed significant changes in bacterial community diversity and structure when applying nZVI to soils as well as Kirschling et al. (2010) in contaminated groundwater using a DGGE approach, whereas Barnes et al. (2010) found no impact of nZVI in river-water bacterial communities.

When re-cultivated in fresh media without nZVI (step 3), the temporary inhibition in nitrate reduction observed for cultures previously exposed to nZVI could also correspond to an adaptation period in the opposite way, due to the absence of nZVI in fresh substrate. Lag phase represents the earliest and most poorly understood stage of the bacterial growth cycle (Rolfe et al., 2012). In bacterial growth models, lag phase is generally described as an adaptation phase of the bacterial community when introduced in a new environment. In our case, the changing parameter is the absence of nZVI in the fresh culture media (lower oxidative stress, lower Fe concentrations). Bacterial growth after this lag phase is expected to be exponential (Rolfe et al., 2012). As the conditions were the same for the cultures without nZVI, it therefore seems logical that no lag phase was observed. As previously shown (Rolfe et al., 2012), high concentrations of iron could be observed in bacterial cells during lag phase, and these concentrations were associated with transient sensitivity to oxidative stress. Moreover, it was shown that toxicity of nZVI is growth phase dependent (Chaithawiwat et al., 2016). A new exposition to nZVI during this lag phase (corresponding to a resilience period for the bacterial communities), would possibly have been lethal for the bacterial community.

Surprisingly, toxicity was higher for lower nZVI concentrations. Even if DLS analyses did not enable to highlight significant differences between the different concentrations in terms of mean sizes of NP aggregates and NP size sorting, trends were observed: more single nZVI or small aggregates were present in lower nZVI concentrations, and higher nZVI concentrations tend to result in the formation of higher aggregates. Consequently, the increase of NP concentrations enhances their aggregation. These observations are consistent with the literature (Baalousha, 2009). During exposure to nZVI, pH tended to increase, and was always above pH 8.0. For pH > 7, functional groups of bacteria and biofilms are globally deprotonated, and are thus negatively charged (Fein et al., 2001; Borrok and Fein, 2004). The formation of ionic bridges between negatively charged bacterial cells and NP with divalent cations present in solution (e.g., Ca^2+^ and Mg^2+^), could lead to strong interactions between bacterial membranes and nZVI, and thus to toxic effects. The presence of large aggregates could result in a decrease in these interactions, and consequently in the observed decrease in toxicity. The formation of large aggregates in cultures with higher nZVI concentrations decreases the ZVI area per unit volume of liquid (Baalousha, 2009), and could also lead to the observed lower toxicity for higher nZVI concentrations. ROS generated would indeed be lower in the media with lower active ZVI area per volume unit, leading to a lower toxic effect.

As toxicity may be due to different mechanisms, these mechanisms may differ as a function of nZVI concentrations. At high concentrations, the main toxicity mechanism could be due to the release of ROS. At low concentrations, it could be rather due to cell/NP interactions. There is probably a co-occurrence of these mechanisms in any case, but their relative part could be dependent on nZVI concentrations and subsequent aggregation. As Fajardo et al. (2012) suggested for soils, these results point to a dose and species-specific impact of nZVI applications in groundwater.

## Conclusion

This study aimed to characterize the impact of nZVI addition on a bacterial community grown in controlled conditions from a natural groundwater sample. The impact of nZVI addition was assessed on bacterial abundance, activity (especially denitrifying activity) and community structure. Even if the impact is only temporary on abundance and activity, due to a good resilience/adaptation of the bacterial community, a shift was observed in community structure. Remediation of contaminated groundwater using nZVI could consequently expose the bacterial groundwater community to shifts in their structures, and thus such techniques could have a long-term impact on bacterial communities and by extension on groundwater ecological functions, such as nitrate reduction. It is consequently important to study the impact on the bacterial community structure and on potential metabolic functions of the aquifers, at the long-term and at the field-scale.

## Author Contributions

MCr contributed to the experimental design, carried out experimental work, data analysis, and drafted the manuscript. JH, CJ, and PO contributed to the experimental design, data analysis, and drafting and revising the manuscript. MCh carried out some of the experiments linked to molecular biology in the study.

## Conflict of Interest Statement

The authors declare that the research was conducted in the absence of any commercial or financial relationships that could be construed as a potential conflict of interest.

## Footnotes

1 http://www.ncbi.nlm.nih.gov/blast/Blast.cgi
