# Supplementary material for: Shift in Natural Groundwater Bacterial Community Structure Due to Zero-Valent Iron Nanoparticles (nZVI)
Source: Front Microbiol. 2019 Mar 19;10:533. doi: 10.3389/fmicb.2019.00533 (PMC6436198; doi:10.3389/fmicb.2019.00533)
Supplement: Supplementary file 1 [file Data_Sheet_1.pdf]

Supplementary Material

Shift in natural groundwater bacterial community structure due to Zero-Valent Iron nanoparticles (nZVI)

Marc Crampon*, Catherine Joulian, Patrick Ollivier, Mickaël Charron, Jennifer Hellal

*** Correspondence:** Corresponding Author: m.crampon@brgm.fr

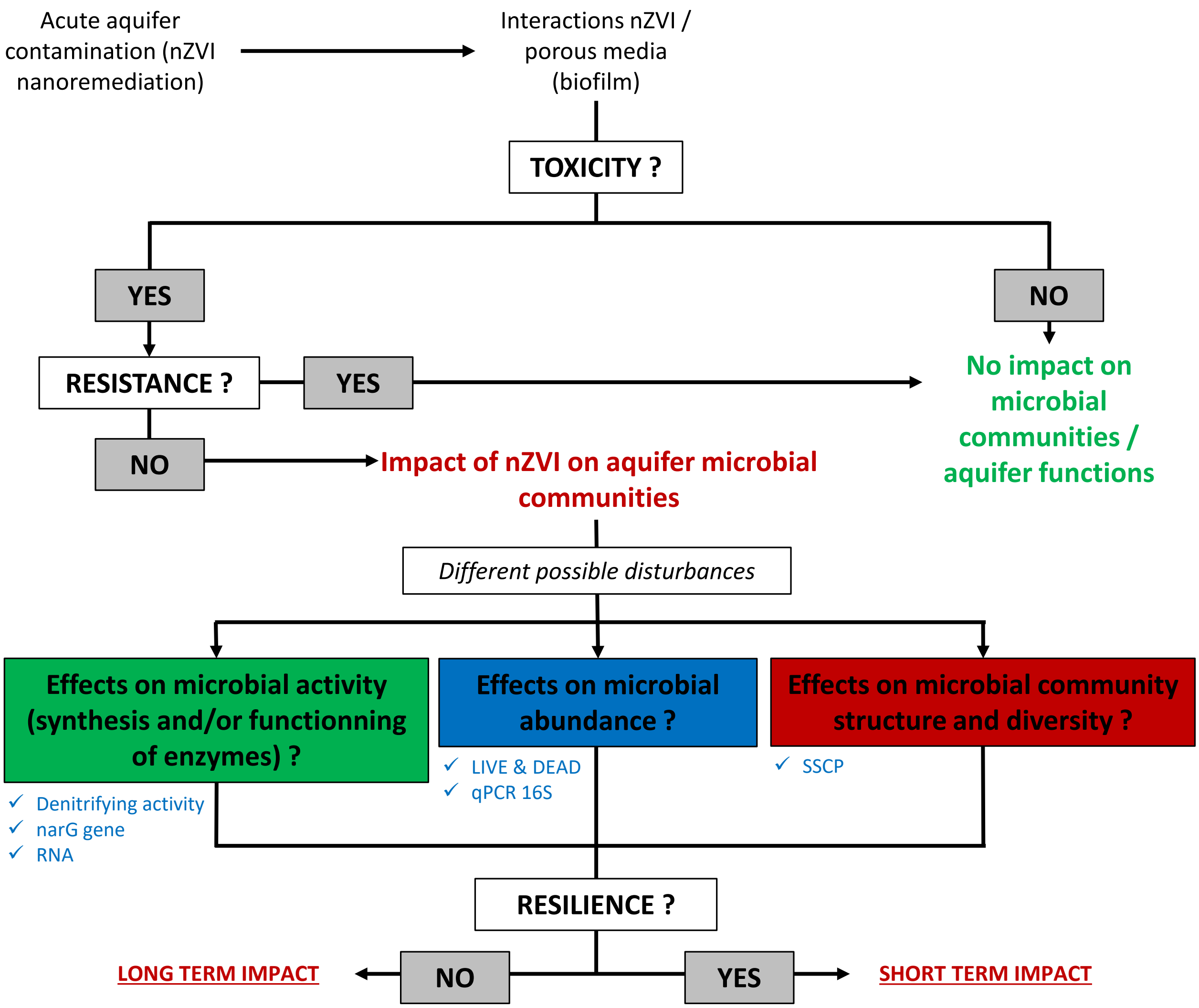

SM1: Response of groundwater bacterial communities to nZVI contamination in the context of a nanoremediation process. The colours (green for activity, blue for abundance and red for structure/diversity) are used throughout the manuscript. Adapted from Simonin and Richaume (2015).

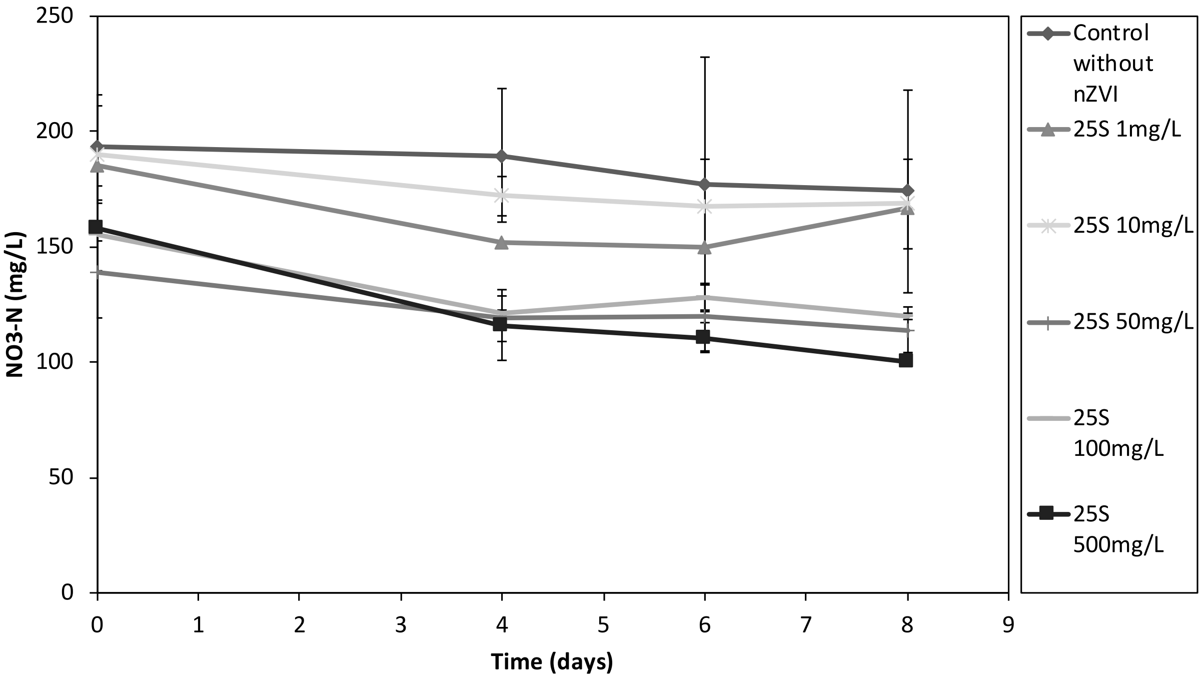

SM2: Results of preliminary experiments on the impact of different nZVI concentrations (1 to 500 mg.L^{-1}) on the reduction of nitrates in sterile conditions

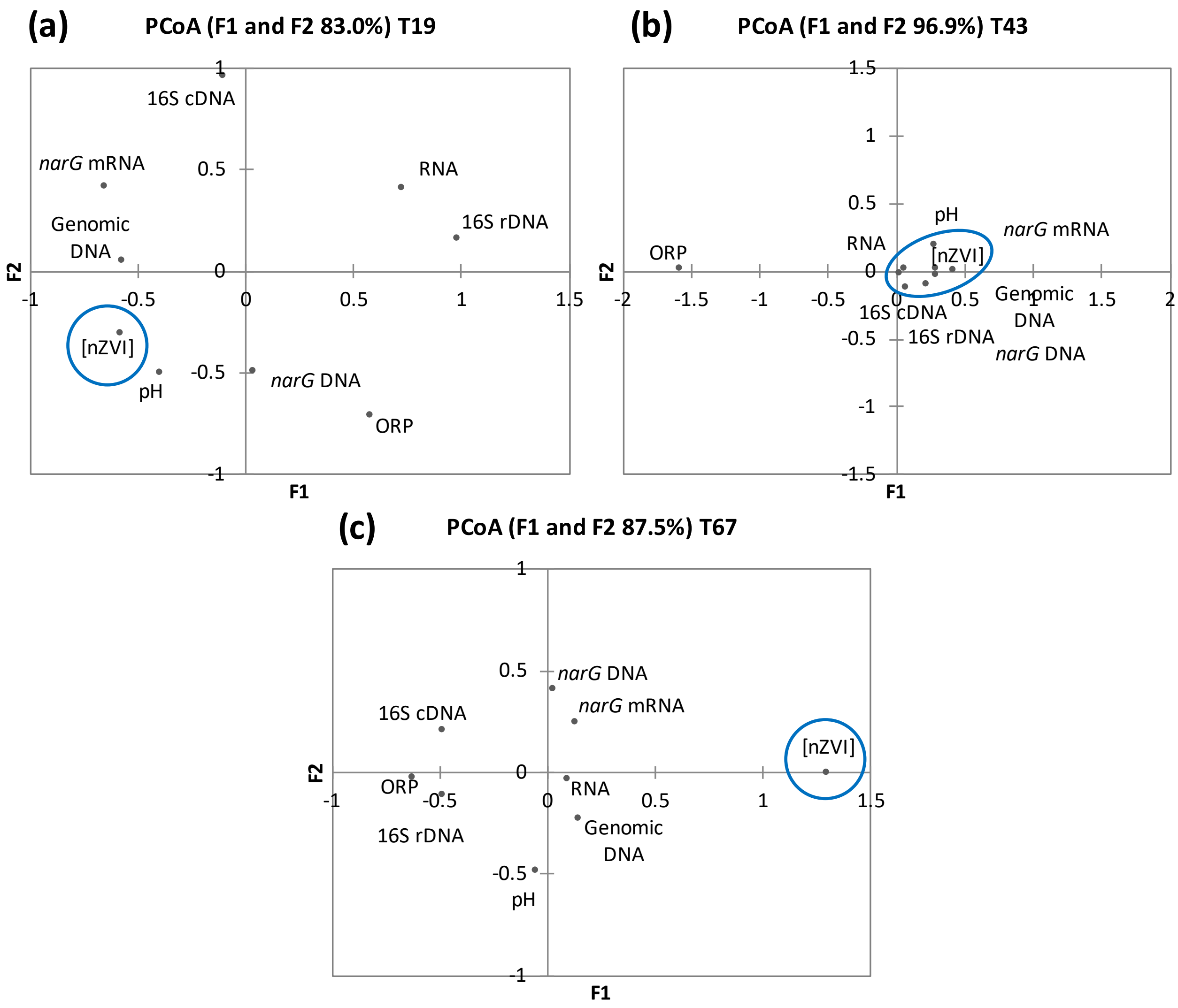

SM3: PCoA analysis of parameters from DNA and RNA analyses concerning abundance and activity of the bacterial community at different times during the experiment for conditions with nZVI (i.e. B, C and D): (a) T19 (before nZVI addition), (b) T43 (24h after nZVI addition) and (c) T67 (48h after nZVI addition). After nZVI addition (b), all studied parameters are closely related to nZVI concentration, in accordance with the toxic impact observed on the bacterial community with individual data, except ORP because its value decreased with an increase in nZVI concentrations.

SM4 : Nitrate-reduction ability of known *Pseudomonas* species phylogenetically related to clones retrieved before and after nZVI addition

		Nitrate reduction	A(na)erobic	Reference
Pseudomonas	*Lini*	**Positive**	Facultative	Delorme et al., 2002
Pseudomonas	*migulae*	**Positive**	Facultative	Verhille et al., 1999
Pseudomonas	*panacis*	**Positive**	Facultative	Park et al., 2005
Pseudomonas	*fluorescens*	**Positive**	Facultative	Anzai et al., 2000
Pseudomonas	*veronii*	**Positive**	Facultative	Elomari et al., 1996
Pseudomonas	*extremaustralis*	**Positive**	Facultative	Lopez et al., 2009
Pseudomonas	*chlororaphis*	**Positive**	Facultative	Pinho et al. 2004
Pseudomonas	*mandelii*	**Positive**	Facultative	Dandie et al. 2007
Pseudomonas	*savastanoi*	*Negative*	Strictly Aerobic	Gardan et al., 1992
Pseudomonas	*kilonensis*	*Negative*	Facultative	Sikorski et al., 2001
Pseudomonas	*helleri*	*Negative*	Strictly Aerobic	Von Neubeck et al., 2016
Pseudomonas	*jessenii*	*Negative*	Facultative	Verhille et al., 1999
Pseudomonas	*vancouverensis*	**Positive**	Aerobic	Mohn et al., 1999
Pseudomonas	*chengduensis*	**Positive**	Facultative	Tao et al., 2014
Pseudomonas	*stutzeri*	**Positive**	Facultative	Lalucat et al., 2006
Pseudomonas	*"songnenensis"*	*Negative*	Aerobic	Zhang et al., 2015
Pseudomonas	*xanthomarina*	**Positive**	Strictly Aerobic	Romanenko et al., 2005
Pseudomonas	*kunmingensis*	**Positive**	Strictly Aerobic	Xie et al., 2014
Pseudomonas	*knackmussii*	*Negative*	Facultative	Stolz et al., 2007
Pseudomonas	*chloritidismutans*	*Negative*	Facultative	Wolterink et al., 2002

REFERENCES

Anzai, Y., Kim, H., Park, J.Y., Wakabayashi, H., and Oyaizu, H. (2000). Phylogenetic affiliation of the pseudomonads based on 16S rRNA sequence. International Journal of Systematic and Evolutionary Microbiology 50(4), 1563-1589. doi: doi:10.1099/00207713-50-4-1563.

Dandie, C., Burton, D., Zebarth, B., Trevors, J., and Goyer, C. (2007). Analysis of denitrification genes and comparison of nosZ, cnorB and 16S rDNA from culturable denitrifying bacteria in potato cropping systems. Systematic and applied microbiology 30(2), 128-138.

Delorme, S., Lemanceau, P., Christen, R., Corberand, T., Meyer, J.-M., and Gardan, L. (2002). Pseudomonas lini sp. nov., a novel species from bulk and rhizospheric soils. International journal of systematic and evolutionary microbiology 52(2), 513-523.

Elomari, M., Coroler, L., Hoste, B., Gillis, M., Izard, D., and Leclerc, H. (1996). DNA relatedness among Pseudomonas strains isolated from natural mineral waters and proposal of Pseudomonas veronii sp. nov. International Journal of Systematic and Evolutionary Microbiology 46(4), 1138-1144.

Gardan, L., Bollet, C., Ghorrah, M.A., Grimont, F., and Grimont, P. (1992). DNA relatedness among the pathovar strains of Pseudomonas syringae subsp. savastanoi Janse (1982) and proposal of Pseudomonas savastanoi sp. nov. International Journal of Systematic and Evolutionary Microbiology 42(4), 606-612.

Lalucat, J., Bennasar, A., Bosch, R., García-Valdés, E., and Palleroni, N.J. (2006). Biology of Pseudomonas stutzeri. Microbiology and Molecular Biology Reviews 70(2), 510-547.

López, N.I., Pettinari, M.J., Stackebrandt, E., Tribelli, P.M., Põtter, M., Steinbüchel, A., et al. (2009). Pseudomonas extremaustralis sp. nov., a Poly (3-hydroxybutyrate) producer isolated from an antarctic environment. Current microbiology 59(5), 514-519.

Mohn, W.W., Wilson, A.E., Bicho, P., and Moore, E.R. (1999). Physiological and phylogenetic diversity of bacteria growing on resin acids. Systematic and applied microbiology 22(1), 68-78.

Park, Y.-D., Lee, H.B., Yi, H., Kim, Y., Bae, K.S., Choi, J.-E., et al. (2005). Pseudomonas panacis sp. nov., isolated from the surface of rusty roots of Korean ginseng. International Journal of Systematic and Evolutionary Microbiology 55(4), 1721-1724. doi: doi:10.1099/ijs.0.63592-0.

Pinho, D., Besson, S., Brondino, C.D., de Castro, B., and Moura, I. (2004). Copper-containing nitrite reductase from Pseudomonas chlororaphis DSM 50135. Eur J Biochem 271(12), 2361-2369. doi: 10.1111/j.1432-1033.2004.04155.x.

Romanenko, L.A., Uchino, M., Falsen, E., Lysenko, A.M., Zhukova, N.V., and Mikhailov, V.V. (2005). Pseudomonas xanthomarina sp. nov., a novel bacterium isolated from marine ascidian. J Gen Appl Microbiol 51(2), 65-71.

Sikorski, J., Stackebrandt, E., and Wackernagel, W. (2001). Pseudomonas kilonensis sp. nov., a bacterium isolated from agricultural soil. International Journal of Systematic and Evolutionary Microbiology 51(4), 1549-1555. doi: doi:10.1099/00207713-51-4-1549.

Stolz, A., Busse, H.-J., and Kaempfer, P. (2007). Pseudomonas knackmussii sp. nov. International journal of systematic and evolutionary microbiology 57(3), 572-576.

Tao, Y., Zhou, Y., He, X., Hu, X., and Li, D. (2014). Pseudomonas chengduensis sp. nov., isolated from landfill leachate. International Journal of Systematic and Evolutionary Microbiology 64(1), 95-100. doi: doi:10.1099/ijs.0.050294-0.

Verhille, S., Baida, N., Dabboussi, F., Hamze, M., Izard, D., and Leclerc, H. (1999). Pseudomonas gessardii sp. nov. and Pseudomonas migulae sp. nov., two new species isolated from natural mineral waters. Int J Syst Bacteriol 49 Pt 4, 1559-1572. doi: 10.1099/00207713-49-4-1559.

von Neubeck, M., Huptas, C., Glück, C., Krewinkel, M., Stoeckel, M., Stressler, T., et al. (2016). Pseudomonas helleri sp. nov. and Pseudomonas weihenstephanensis sp. nov., isolated from raw cow's milk. International Journal of Systematic and Evolutionary Microbiology 66(3), 1163-1173. doi: doi:10.1099/ijsem.0.000852.

Wolterink, A.F., Jonker, A.B., Kengen, S.W., and Stams, A.J. (2002). Pseudomonas chloritidismutans sp. nov., a non-denitrifying, chlorate-reducing bacterium. Int J Syst Evol Microbiol 52(Pt 6), 2183-2190. doi: 10.1099/00207713-52-6-2183.

Xie, F., Ma, H., Quan, S., Liu, D., Chen, G., Chao, Y., et al. (2014). Pseudomonas kunmingensis sp. nov., an exopolysaccharide-producing bacterium isolated from a phosphate mine. International Journal of Systematic and Evolutionary Microbiology 64(2), 559-564. doi: doi:10.1099/ijs.0.055632-0.

Zhang, L., Pan, Y., Wang, K., Zhang, X., Zhang, S., Fu, X., et al. (2015). Pseudomonas songnenensis sp. nov., isolated from saline and alkaline soils in Songnen Plain, China. Antonie van Leeuwenhoek 107(3), 711-721.
